# Supplementary material for: ACCU3RATE: A mobile health application rating scale based on user reviews
Source: PLoS One. 2021 Dec 16;16(12):e0258050. doi: 10.1371/journal.pone.0258050 (PMC8675707; doi:10.1371/journal.pone.0258050)
Supplement: Attachment — Submitted filename: PLOS ONE journal review rev MM.docx [file pone.0258050.s002.docx]

**Original Manuscript ID:** PONE-D-21-09300-R1

**Original Article Title: “**The Influence of User Reviews on Mobile Health Application Rating”

To

The Editor

PLOS ONE

Dear Editor,

Thank you for allowing a resubmission of our manuscript, with an opportunity to address the comments.

We are uploading (a) our point-by-point response to the comments (below) (response to editor), (b) an updated manuscript with yellow highlighting indicating changes, and (c) a clean updated manuscript without highlights (PDF main document).

Best regards,

Biswas et al.

**Concerns of the Editor**

**Concern # 1:**

Please explain the rationale for the development of your rating scale in light of recent research in this area, clearly indicating which problem with existing approaches you are addressing.

**Author response:** Thank you very much for your comment. We have now included the existing mHealth app rating scales in the manuscript and clearly indicated the rationale behind proposing a new mHealth app rating scale.

**Author action:** We updated the manuscript by ….

On pages 4 and 5:

**Concern # 2:**

Please clearly report at the beginning of your methods or results section which the key performance measures were to establish validity and utility of your new system.

**Author response:** Thank you for this comment. The performance measures are included in the revised manuscript.

**Author action:** We updated the manuscript as follows:

**In Tool and Rating Correlation subsection on Page 12 and Page 13**

**Concern # 3:**

your manuscript to include a comparison of the performance/utility of your scale compared. Please modify to existing alternatives. Please note that PLOS ONE requires that experiments, statistics, and other analyses must be performed to a high technical standard and described in sufficient detail to allow for reproducibility of the study (<http://journals.plos.org/plosone/s/criteria-for-publication#loc-3>). To demonstrate the performance of the scale, we would expect comparisons to be drawn between existing state-of-the-art methods.

**Author response:** Thank you very much for your guidance. The performance metrics used in evaluating the proposed mHealth app rating scale is included in the revised manuscript. However, we have not included any comparison between the proposed mHealth app rating scale and the existing ones as the evaluation criterion in each scale is different, and the comparison will not be fair.

**Author action:** We updated the manuscript to reflect this.

**In Tool and Rating Correlation subsection on Page 12 and Page 13 shows the performance metrics used in validating the proposed scale.**

In Figure 11 on Page 16 shows comparison between traditional and fuzzy based app rating.

**In Results and Discussion on Page 15**

In the Conclusion section on page 18,
