# Supplementary material for: ACCU3RATE: A mobile health application rating scale based on user reviews
Source: PLoS One. 2021 Dec 16;16(12):e0258050. doi: 10.1371/journal.pone.0258050 (PMC8675707; doi:10.1371/journal.pone.0258050)
Supplement: Attachment — Submitted filename: PONE-D-21-09300-Authors-reply.docx [file pone.0258050.s003.docx]

PONE-D-21-09300

The Influence of User Reviews on Mobile Health Application Rating

Editor Comments:

------------------------

The idea of the paper is interesting. There are some major concern of reviewer 2 and 3. The paper need improvement. The paper also contain some grammatical and spelling mistakes. Proofreading of paper is required.

Author response: Thank you. We have addressed all the concerns of reviewers. In addition, the manuscript has been carefully proofread to eliminate grammatical and spelling mistakes.

Reviewer # 1

------------------

Response to the Reviewers’ Concerns Reviewer # 1

Comment # 1:

This paper presents an Artificial Intelligence (AI)-enabled Health app rating tool which takes multidimensional measures such as star rating, user’s review and features declared by the developer to generate app rating. The paper is well written and can be accepted for publication.

Author response: Thank you so much for your encouragement. It is appreciated.

Reviewer # 2

-------------

Response to the Reviewers’ Concerns Reviewer # 2

Comment # 1:

This study applies AI text mining technique to develop more comprehensive understanding of users' feedback based on an array of factors, determining the mHealth app ratings. Overall, the paper is well written and easy to follow.

There are some minor issues to be addressed:

Concern # 1:

Main limitations need to be also explained/discussed

Author response: The manuscript has been updated by including the scopes / limitations of this research which has been explained in the lines 13 to 83 on pages 2 and 3.

Concern # 2: why a fuzzy logic has been employed? further motivations are needed

Author response: The revised manuscript is updated by including the discussion regarding Fuzzy logic Controller for information fusion in lines 84 to 91 on page 3

Concern # 3:

Future works are limited. The author should further discuss this section. In the future, explainable AI aspects should be addressed in order to give a deeper understanding of the proposed system. In this sense the authors can refer to:

\# A novel explainable machine learning approach for EEG-based brain-computer interface systems;

\# Towards multi-modal causability with Graph Neural Networks enabling information fusion for explainable AI

Author response: Thank you so much. In the revised manuscript, the future work section has been rewritten which can be seen in lines 549 to 554 on page 18.

Also, both relevant references have been included in the revised manuscript in the reference section on page 25.

Comment # 2 Generally, this work is important and with the modifications this can be a really useful archival work for the international community. Congrats to the authors and this reviewer hopes that the suggestions help to improve the paper.

Author response: Thank you for your encouragement.

Reviewer # 3

-------------

Response to the Reviewers’ Concerns Reviewer # 3

Comment # 1: This manuscript presents a novelty ranking of mobile health applications. This ranking is based on the user's star ranking, users' text review, clinical approval, UI design, functionality, security, and privacy. Results suggest a competitive performance of the proposed ranking compared against app and expert rating.

Author response: Thank you very much.

Concern # 1: Review some misspelling words (invesgitation on page 2). I recommend reading the entire document to avoid this writing mistake.

Author response: Thank you for pointing out this issue. The manuscript has been thoroughly proofread to eliminate any typos and grammatical errors.

Concern # 2: Algorithm 1 is located several pages below its mention. I suggest including it below of its reference.

Author response: Thank you. Algorithm 1 has now been placed on page 11 and the references to the algorithm are mentioned afterwards (Lines 439-460).

Concern # 3: Into the manuscript are mentioned Questionaries Q1-Q4 for Clinical Approval measure. Please, include a detailed comment about this because it looks like a disconnected element. I understand that they are used for getting the clinical	approval	value, but	the	questionaries	are	not	described.

Author Response: Thank you. We have now included description of the Questions Q1-Q4 in the Approval and Certification section on page 13 (Lines 507-512).

Concern # 4: Are mentioned five scales for ranking apps: THESIS, MARS, Brief DISCERN, uMARS, and ORCHA-24. Compared with these, how is your proposal? is better, worst, or similar? Please provides a comparison with these methods.

Author response: Thank you for pointing out this issue. We have added the performance comparison of different scales in terms of Intra-class Correlation Coefficient (ICC). However, the performance of Brief DISCERN [50] could not be included in this comparison as it used a different evaluation scale. We updated the manuscript by including Figure 11 (D) on page 17,

And the related description in lines 602- 608 on the same page.
