# Supplementary material for: ACCU3RATE: A mobile health application rating scale based on user reviews
Source: PLoS One. 2021 Dec 16;16(12):e0258050. doi: 10.1371/journal.pone.0258050 (PMC8675707; doi:10.1371/journal.pone.0258050)
Supplement: Attachment — Submitted filename: Response to reviewers.docx [file pone.0258050.s004.docx]

**Original Manuscript ID:** PONE-D-21-09300-R2

**Original Article Title: “**The Influence of User Reviews on Mobile Health Application Rating”

To

The Editor

PLOS ONE

Dear Editor,

Thank you for allowing a resubmission of our manuscript, with an opportunity to address the comments given by the reviewers. The manuscript has been carefully proofread to eliminate grammatical and spelling mistakes. In addition, some of the sections are rewritten to bring coherence and improve the fluency of the article. The yellow highlighting indicates changes in the revised manuscript. We are uploading

(a) our point-by-point response to the comments (response to Reviewer),

(b) an updated manuscript with yellow highlighting indicating changes, and

(c) a clean updated manuscript without highlights (PDF main document).

Best regards,

Biswas et al.

**Concerns of the Reviewer 3**

**Concern # 1: :** In Figure 11(D), a comparison among the scale against the proposal is detailed, but there is no discussion of what means these values concerning ICC. How is the proposal behavior based on this comparison?. Therefore, it is essential to mention it within the text.

**Author response: Thank you very much. In Figure 11 (D) a comparison among the scale has been presented. The discussion is now included in the revised manuscript.**

**Author action:** We updated the manuscript by including the following

**Concern # 2:** In line 515, the authors use the "Fig." term, and the lines above use "Figure" please uses the same terminology in all documents.

**Author response: Thank you very much. We have replaced Fig. by Figures (on line 538) in the revised manuscript.**

**Author action:** The changes are shown in the screenshot given above.
